# Expanding the Druggable Space of the LSD1/CoREST Epigenetic Target: New Potential Binding Regions for Drug-Like Molecules, Peptides, Protein Partners, and Chromatin

**DOI:** 10.1371/journal.pcbi.1003158

**Published:** 2013-07-18

**Authors:** James C. Robertson, Nate C. Hurley, Marcello Tortorici, Giuseppe Ciossani, Maria Teresa Borrello, Nadeem A. Vellore, A. Ganesan, Andrea Mattevi, Riccardo Baron

**Affiliations:** 1 Department of Medicinal Chemistry, College of Pharmacy, The University of Utah, Salt Lake City, Utah, United States of America; 2 Department of Biology and Biotechnology, University of Pavia, Pavia, Italy; 3 School of Pharmacy, University of East Anglia, Norwich Research Park, Norwich, United Kingdom; University of Houston, United States of America

## Abstract

Lysine specific demethylase-1 (LSD1/KDM1A) in complex with its corepressor protein CoREST is a promising target for epigenetic drugs. No therapeutic that targets LSD1/CoREST, however, has been reported to date. Recently, extended molecular dynamics (MD) simulations indicated that LSD1/CoREST nanoscale clamp dynamics is regulated by substrate binding and highlighted key hinge points of this large-scale motion as well as the relevance of local residue dynamics. Prompted by the urgent need for new molecular probes and inhibitors to understand LSD1/CoREST interactions with small-molecules, peptides, protein partners, and chromatin, we undertake here a configurational ensemble approach to expand LSD1/CoREST druggability. The independent algorithms FTMap and SiteMap and our newly developed Druggable Site Visualizer (DSV) software tool were used to predict and inspect favorable binding sites. We find that the hinge points revealed by MD simulations at the SANT2/Tower interface, at the SWIRM/AOD interface, and at the AOD/Tower interface are new targets for the discovery of molecular probes to block association of LSD1/CoREST with chromatin or protein partners. A fourth region was also predicted from simulated configurational ensembles and was experimentally validated to have strong binding propensity. The observation that this prediction would be prevented when using only the X-ray structures available (including the X-ray structure bound to the same peptide) underscores the relevance of protein dynamics in protein interactions. A fifth region was highlighted corresponding to a small pocket on the AOD domain. This study sets the basis for future virtual screening campaigns targeting the five novel regions reported herein and for the design of LSD1/CoREST mutants to probe LSD1/CoREST binding with chromatin and various protein partners.

## Author Summary

Protein dynamics plays a major role in determining the molecular interactions available to molecular binding partners, including druggable hot spots. The LSD1/CoREST complex is one of the most relevant epigenetic targets discovered and was shown to be a highly dynamic nanoscale clamp using molecular dynamics simulations. The general relationship between LSD1/CoREST dynamics and the molecular sites available for non-covalent interactions with an array of known binding partners (from relatively small drug-like molecules and peptides, to larger proteins and chromatin) remains relatively unexplored. We employed an integrated experimental and computational biology approach to effectively capture the nature of non-covalent binding interactions available to the LSD1/CoREST nanoscale complex. This ensemble approach relies on the newly developed graphical visualization by Druggable Site Visualizer (DSV) that allows treatment of large-size protein configurational ensembles data and is freely distributed to the public and readily transferable to other protein targets of pharmacological interest.

## Introduction

**Figure 1:**
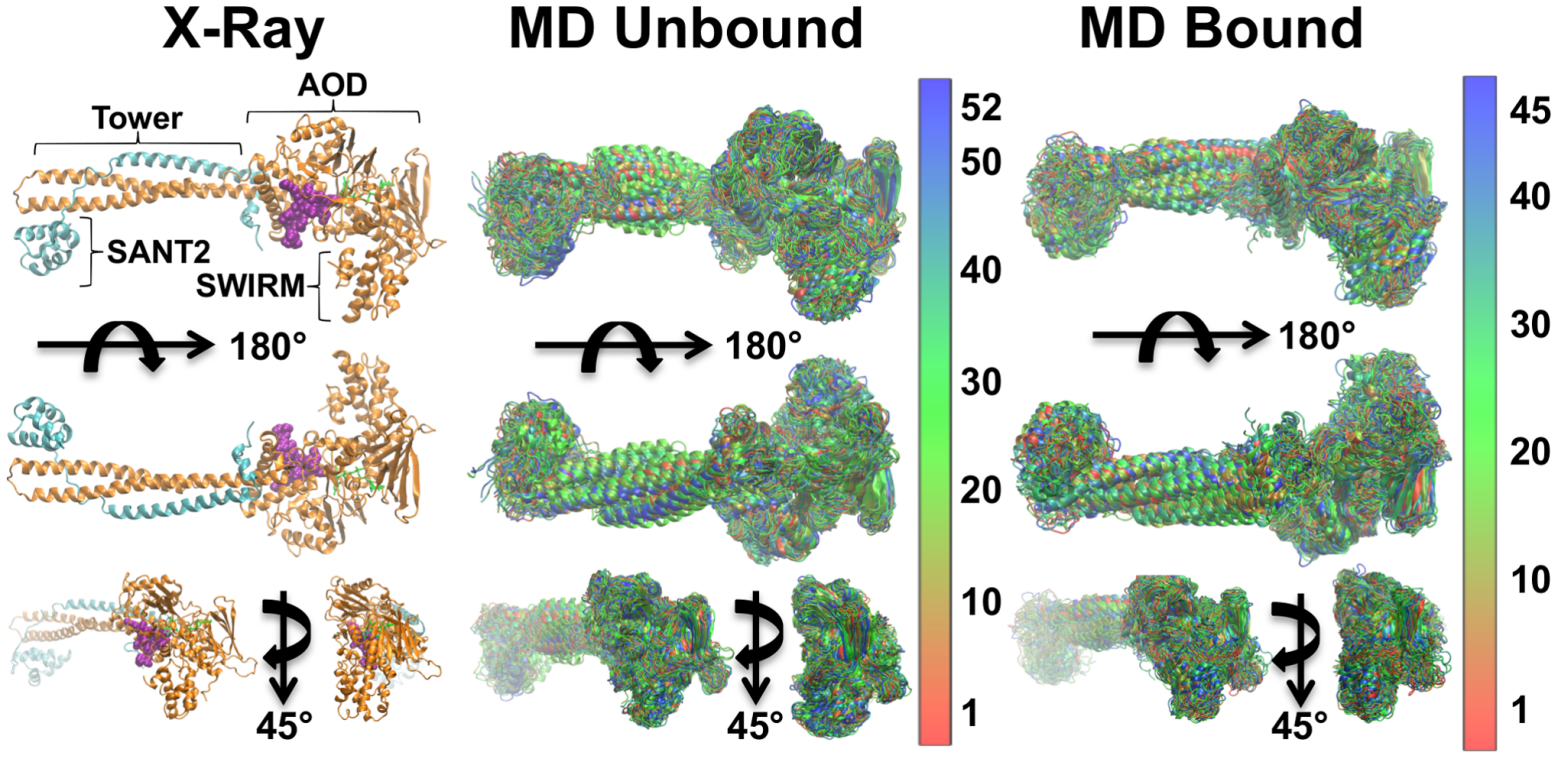
Comparison of LSD1/CoREST X-ray structure and heterogeneous conformations from conformational clustering of molecular dynamics trajectories. Left column: X-ray structure of LSD1/CoREST bound to the H3-histone N-terminal tail (PDB ID: 2V1D); LSD1 (orange cartoons), CoREST (cyan cartoons), H3-tail (purple spheres), and the FAD cofactor (green tubes) are highlighted. LSD1/CoREST has a well-characterized amine oxidase domain (AOD) that binds the H3-histone N-terminal tail and demethylates the fourth lysine residues of the H3-histone N-terminal tail. Connected to the AOD is the SWIRM domain crucial for substrate recognition [26]. A unique feature of LSD1 is the Tower domain that serves as interface for associating with CoREST, and is required for nucleosome binding. Middle column: MD centroids of the reduced unbound conformational ensemble. Right column: MD centroids of the reduced H3-histone N-terminal tail-bound conformational ensemble. MD centroids are color coded from red (high centroid rank) to blue (low centroid rank).

Lysine specific demethylase-1 with its corepressor protein CoREST (LSD1/CoREST) has emerged as one of the most promising epigenetic targets in drug discovery and design [1]. LSD1/CoREST is widely investigated for its expanding biological roles in cancer, neurodegeneration, and viral infection [2]–[7]. The precedence for drugging chromatin modifying epigenetic targets was established with FDA approval of vironostat and romidepsin, antineoplastic epigenetic drugs that target histone deacetylases [8]–[10]. However, no promising therapeutics that target LSD1/CoREST have emerged to date. A few LSD1 inhibitors have been reported [6] but they display modest activity, have non-ideal medicinal chemistry features due to their polycationic nature [11], [12] or are poorly selective covalent inhibitors that bind to FAD in the H3-histone N-terminal tail-binding pocket (Figure 1) [13]–[15]. Alternatively, short peptide sequences have been recently designed to bind with affinities comparable to those displayed by the natural H3-histone substrate [16] and are inspiring the development of lead compounds. Recently, our group proposed that druggable regions beyond the AOD active site (Figure 1) might hold the key to developing pharmacologically relevant inhibitors by an allosteric mechanism revealed by extended molecular dynamics (MD) simulations [17], [18]. Moreover, these new druggable regions could target protein-protein interactions necessary to the formation of multi-protein complexes [19]–[25] and/or prevent LSD1/CoREST from binding to the nucleosome [18], [26].

Multiple solvent crystal structures (MSCS) is an experimental technique that can probe favorable binding regions for small molecular fragments on protein surfaces. Still, only a reduced number of protein crystals are suited for such experiments because the conditions for MSCS can interfere with crystallization. This limitation highlights the importance of developing reliable computational techniques that quickly and accurately identify potential binding hot spots on a protein receptor. FTMap [27] and SiteMap [28], [29] are two algorithms that were successfully and independently developed to predict druggable hot spots. In order to investigate protein druggability while effectively including receptor dynamics, conformational clustering analysis has been shown to generate reduced receptor configurational ensembles with significant computational timesaving [30]–[33]. Thus far, ensemble-based approaches have often employed clustering algorithms to select only a handful of dominant receptor MD centroids, which are the most representative structures extracted from a conformational clustering analysis, but this poses the general question whether a few most dominant structures are sufficient to capture more ephemeral states of the receptor, which could contribute to important mechanistic steps such as the opening of transient cavities available for binding. Nichols et al. highlighted this problem in the context of blind virtual screening through ligand docking to MD generated receptor structures [34], [35].

In this study, we took a complete-ensemble approach by effectively including all the most relevant MD centroids in addition to available X-ray structures to probe the druggable space of the dynamic LSD1/CoREST epigenetic target (Figure 1). A reduced number of tens of MD centroids allows effectively eliminating redundant information and efficient computational analysis. The entire LSD1/CoREST protein complex was investigated using the independent algorithms FTMap and SiteMap so that previously uncharacterized hot spots could be identified. The newly developed Druggable Site Visualizer (DSV) software tool was used to inspect favorable binding regions. The resultant computational predictions were compared with the available experimental data including X-ray crystallography experiments that used small peptides to investigate protein-protein interactions on the LSD1/CoREST surface. The co-crystallized Pro-Leu-Ser-Phe-Leu-Val peptide in a novel, predicted binding site on LSD1/CoREST shows the strength of the methods hereby presented.

## Materials and Methods

### Molecular Dynamics Simulations

The molecular systems and simulations used in this study were previously described [17], [18]. The atomic coordinates from the structure by Yang et al. (PDB ID: 2IW5; 2.6 Å resolution) [26] were used to initialize a 500 ns run of LSD1/CoREST. A second 500 ns run of LSD1/CoREST bound to the H3-histone N-terminal tail (16 residues) was initialized using the peptide substrate coordinates by Forneris et al. (PDB ID: 2V1D; 3.1 Å resolution) [36]. Standard preparation, minimization, heat-up, and equilibration procedures were performed using GROMACS (version 4.5.4) compiled in double precision [37], [38], the GROMOS 53A6 force field parameter set [39], the compatible SPC water model [40], and compatible ion parameters [41]. 50,000 MD snapshots were extracted every 10 ps from each trajectory and used for analysis.

### Conformational Clustering

An RMSD-based conformational clustering algorithm was used to extract reduced unbound and H3-bound configurational ensembles [42] as implemented in the GROMACS g_cluster program [37], [38]. The snapshots from each trajectory were aligned to each other by least-square fitting [43] of the C^α^ atoms of key residues from the amine oxidase domain (Pro171-Glue427 and Ser517-Lue836). Conformational clustering was performed on all atoms of these residues by scanning a wide range of RMSD similarity thresholds, and the final choice was made by employing a similarity threshold of 2 Å. See the Results section for a detailed discussion of the conformational clustering analysis.

### Druggability Site Mapping

Prior to the mapping calculations each structure was prepared using the Protein Preparation Wizard utility from Schrödinger [44], [45]. Water molecules were removed when present and hydrogen atoms added to reproduce a neutral apparent pH. The position of all hydrogen atoms was energy minimized using the OPLS 2005 force field [46]. The FTMap and SiteMap alternative computational approaches were used to search for favorable binding regions on LSD1/CoREST structures. The FTMap algorithm samples an order of 10^9^ docked poses for 16 small molecule probes using Fast Fourier Transforms. The docked probes are scored and reduced to sets containing the top 2,000 poses for each probe. After minimization the probes are rescored and clustered using a 3-Å cutoff. The SiteMap algorithm generates site points on a grid surrounding the receptor van der Waals surface (0.35 Å grid 3D resolution in our study). Site points sheltered in a pocket or cleft of the protein are retained while points left exposed to solvent are eliminated; the criteria for retaining a site point is determined by the ratio of the squares of the distance of site points to a protein receptor atom and the van der Waals radius of that receptor atom being less than the default value of 2.5 [29]. The remaining site points that have neighbors in close proximity are grouped into SiteMap sites. A probe simulating a water molecule explores each site and characterizes the sites based on van der Waals and electrostatic potentials. Contour maps of each site are generated that describe the binding characteristics of the site. Apart from grid resolution, the SiteMap default settings were employed in all cases and sites were merged with the receptor into a single PDB file for analysis.

### Graphical Modeling and Analysis

**Figure 2:**
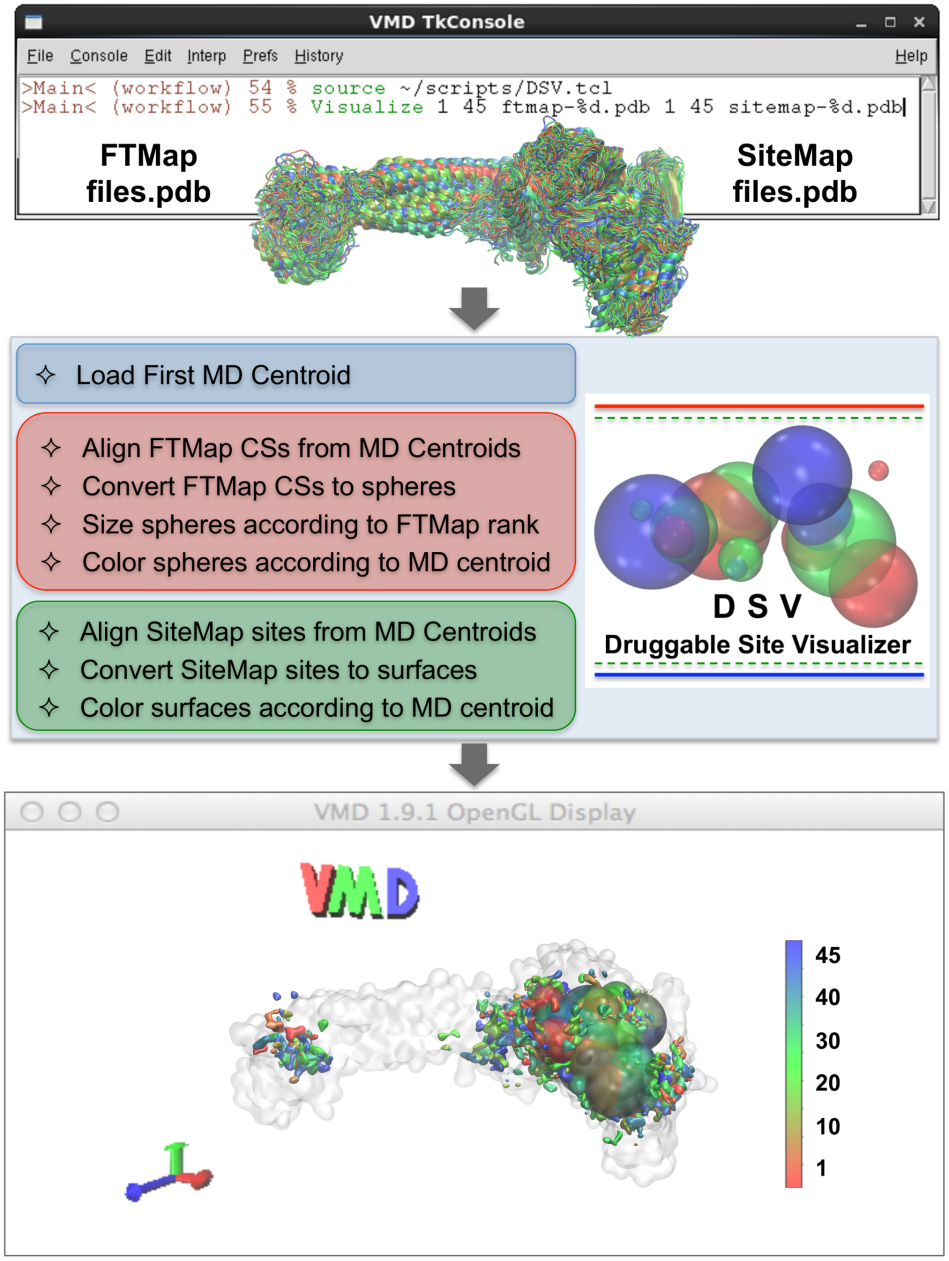
Druggable Site Visualizer (DSV) workflow and graphical interface. The DSV *Visualize* function allows the user to easily view predicted binding sites on multiple receptor structures by mapping FTMap Consensus Sites (CSs) and SiteMap sites on a receptor and displaying the results with Visual Molecular Dynamics (VMD). DSV automated processing of an ensemble of structures displays the predicted binding sites onto a representative user-defined reference structure (in this case the top ranked MD centroid). After sourcing the DSV script, a single text on the command line instructs DSV to load the reference structure, align the remaining centroids to the reference, and then display FTMap CSs as spheres and SiteMap sites as surfaces. The spheres are sized according to CS rank and colored according to their centroid rank. The SiteMap surfaces are also colored according to centroid rank.

The Druggable Site Visualizer (DSV) software was developed for this work as a plugin for graphical modeling with Visual Molecular Dynamics (VMD) [47]. Figure 2 summarizes the DSV workflow and the underlying automated steps that remain blind to the user. The DSV function *Visualize* takes FTMap and SiteMap output in PDB file format and processes it for convenient and data-rich visualization. *Visualize* employs as arguments either a single receptor structure or an ensemble of structures; the latter scenario is subsequently described and used in this work for processing the reduced MD ensembles. The user loads a first PDB structure through DSV and a QuickSurf representation is created. Then the remaining structures with FTMap and SiteMap information are loaded as DSV performs their automated alignment to the first reference structure. DSV converts FTMap consensus sites (CSs) to spheres centered about the geometric midpoint of each CS and sized according to CS rank (largest sphere corresponding to highest ranking CS). This graphical approach was inspired by previous work by Ivetac and McCammon [32] and automated in DSV. DSV colors such FTMap spheres corresponding with the rank of the MD centroid they correspond to (color coding goes from red for highest-ranking MD centroids to blue for lowest ranking MD centroids where rank is determined by population of the MD cluster from which the centroid was extracted by conformational clustering). In parallel, DSV *Visualize* converts the SiteMap sites to isosurface representations colored according to their MD centroid rank. By default, all of the FTMap spheres and SiteMap surfaces are displayed on the first-loaded reference structure.

For graphical purposes the user makes some system dependent, arbitrary decisions. Typical user-defined inputs are:

- The alignment parameters for aligning to the first structure
- The number of FTMap CSs to display
- The sphere radius for FTMap CSs
- The surface isovalue (iValue) to set surface density for SiteMap sites
- The coloring scheme for FTMap spheres and SiteMap sites

In this work the number of CSs displayed for each system are specified in the text and figure captions, LSD1/CoREST structures were aligned based on the C^α^ atoms of all protein residues, the largest sphere radius was set equal to the number of spheres displayed (in Å), and the iValue was set to the default value 0.5.

Another automated feature of DSV is the *Select-residues* function. This function may work with a single receptor structure or an ensemble of structures that contain FTMap and SiteMap output. The latter scenario is subsequently described and used in this work for identifying residues defining new druggable regions as described in the Discussion section. The first PDB reference structure file is loaded through DSV and a NewCartoon representation of the protein receptor is produced. Subsequent structures are loaded through DSV and aligned to the initial reference structure, following an identical procedure described above for the *Visualize* function. *Select-residues* then loops through all MD centroids and selects residues within 3 Å of FTMap CSs and produces licorice representations of the residues on the first structure while removing duplicate occurrences of residues across the ensemble of MD centroids. A licorice representation of residues is created for all residues within 3 Å of SiteMap sites while eliminating redundancy. At the last step, a third representation is created that shows residues in licorice representations for residues within 3 Å of both FTMap and SiteMap sites. For graphical purposes the user inputs some system-dependent decisions. Examples of user-defined inputs in the first release of DSV are:

- The alignment parameters for aligning to the first structure
- The distance to FTMap CSs or SiteMap sites for residue selection
- The receptor and selected residues representations

The first release of DSV (version 1.0) can be freely downloaded at the software tools web page of the Baron lab, currently: http://barongroup.medchem.utah.edu/tools.

### X-Ray Crystallography Experiments

The crystallographic data and three-dimensional structure of LSD1/CoREST bound to the peptide Pro-Leu-Ser-Phe-Leu-Val were described before [16] (PDB ID: 3ZMV). Briefly, the peptide complex was obtained by crystal soaking in solutions consisting of 1.6 M sodium/potassium tartrate, 100 mM N-(2-acetamido)-2-iminodiacetic acid pH 6.5, 10% (v/v) glycerol, and 2–5 mM peptide for 3 h. X-ray diffraction data were collected at 100 K at the Swiss Light Source (Villigen, Switzerland). Data processing and refinement were carried out using programs of the CCP4 package [48].

## Results

The reduced ensembles obtained from conformational clustering contained 52 (unbound) and 45 (H3-bound) MD centroids. Figure 1 shows the MD centroids sorted according to their cluster rank as visualized by Druggable Site Visualizer (DSV). The top-ranking clusters contained 11,643 (unbound) and 10,995 (H3-bound) MD snapshots whereas four (unbound) and three (H3-bound) MD clusters were singly populated. Overall, this result was consistent with the general observation of a moderate decrease in LSD1/CoREST flexibility upon H3-histone binding [17], [18] (Figure 1). Note that this study employed all the MD centroids in each (unbound or H3-bound) reduced ensemble, to account as well for transient and more rare MD snapshots. It is therefore different from previous closely related approaches (e.g. see Refs. [32], [33] that focused the analysis on the most dominant MD centroids only).

**Figure 3:**
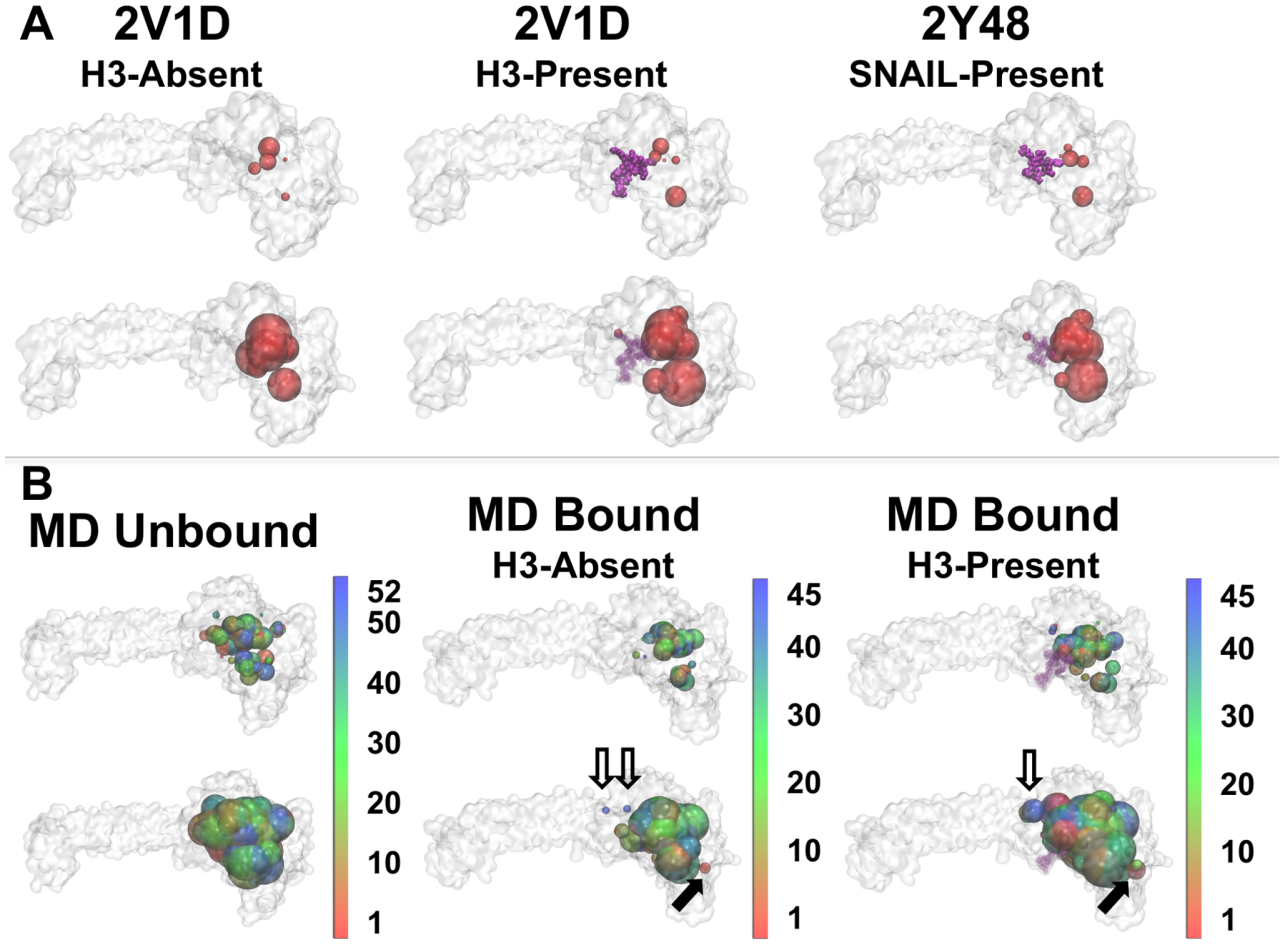
Druggable Site Visualizer (DSV) mapping of the top FTMap consensus sites (CSs). FTMap CSs are shown as red spheres on X-ray structures (**Panel A**) and as spheres colored according to representative MD centroids (**Panel B**). In each panel, the top row displays the top five CSs reported by FTMap; the bottom row displays the top 10 CSs. DSV displays sphere size correlated to FTMap CS rank. For graphical purposes, the FTMap CSs from all representative MD centroids are mapped onto the structure of the highest ranked MD centroid. If present during the FTMap mapping calculations, the H3-histone N-terminal tail (present in 2V1D and MD Bound) and the SNAIL1 N-terminal peptide (present in 2Y48) are highlighted as purple spheres. The H3-histone N-terminal tail and SNAIL1 N-terminal peptide were rendered more transparent when there was overlap with FTMap spheres. Solid arrows highlight new CSs at the AOD/SWIRM interface not observed in the X-ray structures and hollow arrows highlight new CSs at the AOD/Tower interface not observed in the X-ray structures (cf. 2V1D H3-Absent with MD Bound H3-Absent and 2V1D H3-Present with MD Bound H3-Present).

Druggability mapping was first explored using available X-ray structures of the LSD1/CoREST complex. Results based on X-ray structures of LSD1/CoREST bound to the H3 (PDB code 2V1D [36]) and SNAIL (PDB code 2Y48 [49]) N-terminal peptides were mapped with DSV for the five highest-ranking FTMap CSs (Figure 3A, top row) and the 10 highest-ranking FTMap CSs (Figure 3A, bottom row). Druggability mappings of these structures were performed both in the absence (first column) and presence (second and third columns) of the peptide ligands. In all cases, the most likely druggable region picked by FTMap was clearly the well-known H3-pocket. The FAD cofactor pocket was also similarly favored (Figure S1). This result confirmed that new favorable regions were found independently of which X-ray structure was employed, and independently of which peptide substrates occupied the H3-binding site. The observed ability of FTMap to blindly predict favorable LSD1/CoREST sites for non-covalent binding of peptide ligands or of the FAD cofactor confirmed analogous successes recently reported for different protein receptors [27], [50], [51].

After achieving confidence in FTMap accuracy on the LSD1/CoREST complex, druggability mapping was investigated using complete reduced MD ensembles obtained through conformational clustering of each of our 500 ns MD simulations to evaluate the effects of LSD1/CoREST dynamics on the 3D druggable space. Figure 3B shows the five highest-ranking FTMap CSs (top row) and the 10 highest-ranking FTMap CSs (bottom row) on the MD reduced ensembles (Figure 1). The CSs from the unbound and bound reduced ensemble predicted that the H3-pocket and FAD cofactor sites were strongly favorable as observed for the X-ray structures (Figure 3B). However and most important, inclusion of LSD1/CoREST dynamics resulted in remarkably broader predicted druggable regions due to the opening of transient niches and cavities on the protein surface and in the H3-pocket (cf. Figure 3A vs. 3B). Most notably, new CSs were observed at the AOD/SWIRM (solid arrows Figure 3B) and AOD/Tower (hollow arrows Figure 3B) inter-domain interfaces, which widely expanded the druggable regions.

**Figure 4:**
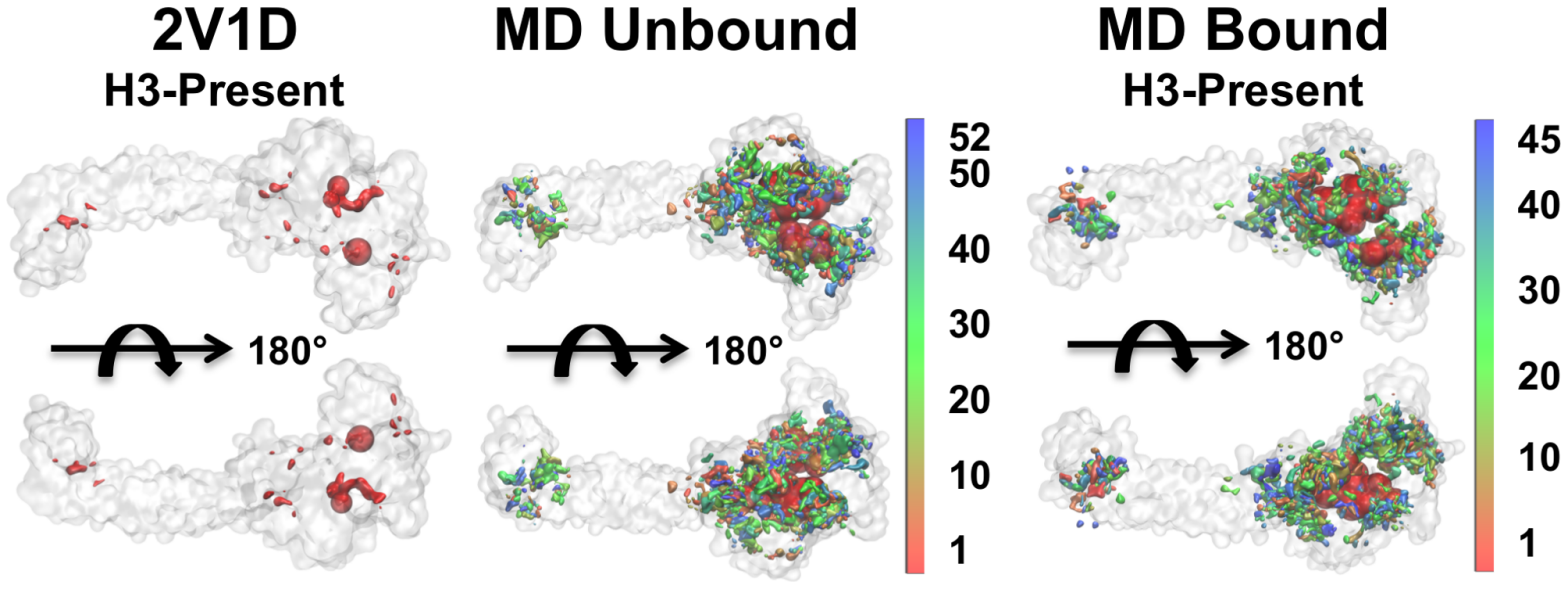
Comparison between FTMap and SiteMap druggability mapping. The top-five FTMap consensus sites (red spheres) and SiteMap sites (surfaces) are displayed as calculated for X-ray and MD structures. DSV *Visualize* was used to display the FTMap and SiteMap results, coloring the SiteMap sites according the MD centroid rank. In all cases SiteMap surfaces overlap with FTMap spheres and predict druggable regions beyond FTMap predicted space.

In addition to performing FTMap calculations on LSD1/CoREST experimental structures and MD reduced ensembles, SiteMap calculations were also performed to explore the druggable space of LSD1/CoREST by means of an alternative, independent algorithm. Figure 4 shows the comparison of the top-five FTMap CSs and SiteMap sites obtained from DSV using the PDB ID 2V1D (H3-histone tail present during FTMap and SiteMap calculations), the unbound MD reduced ensemble, and the H3-bound MD reduced ensemble (H3-histone tail present during FTMap and SiteMap calculations). Consensus between FTMap and SiteMap was expected and largely found, as inferred by the observation that every FTMap sphere overlapped with a predicted SiteMap surface. In all cases, however, the SiteMap sites were also found in regions in which FTMap did not predict favorable sites. Most prominently, SiteMap predicted binding sites in the CoREST-SANT2/Tower region, while FTMap did not. In addition, SiteMap predicted more binding sites along the AOD/Tower inter-domain interface and on the SWIRM domain. Overall, the diverse unbound and H3-bound configurational ensembles led to distinguishable distributions of SiteMap sites on the LSD1/CoREST domains, in line with what was observed using FTMap on the same MD ensembles.

**Figure 5:**
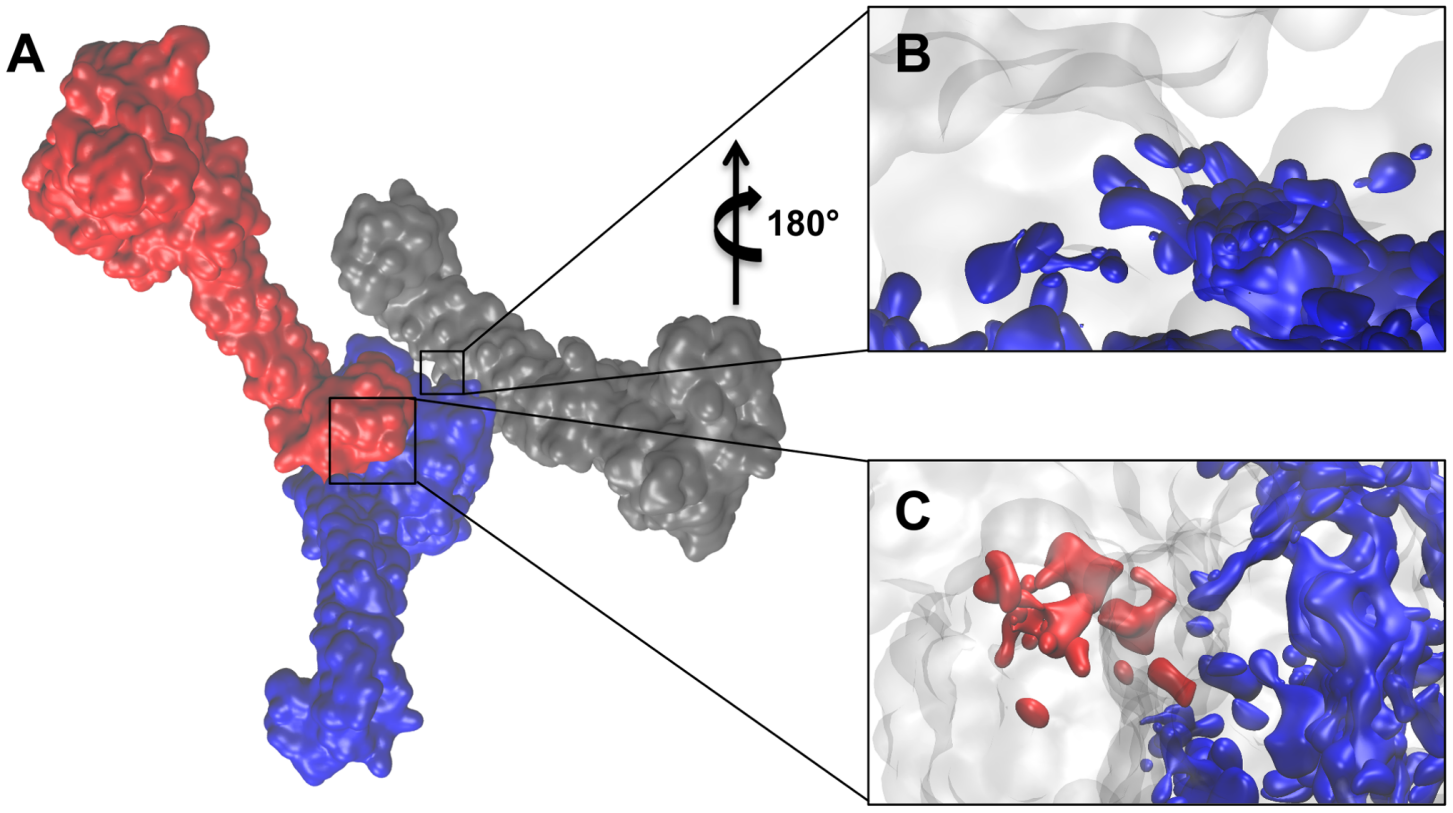
LSD1/CoREST crystal contacts. SiteMap sites on LSD1/CoREST overlap with crystal contact regions. **Panel A** shows the crystal-packing interactions of three LSD1/CoREST molecules represented as red, blue, and grey opaque surfaces. In **Panels B** and **C** LSD1/CoREST are represented as transparent surfaces and SiteMap sites are colored surfaces to correspond with the unit in **Panel A**, e.g. the blue SiteMap sites in **Panel B** originate from the blue LSD1/CoREST in **Panel A**.

Crystal contacts on protein surfaces and computational hot spot prediction have been used to predict protein-protein interactions in the past [52], [53]. We thought to compare the LSD1/CoREST regions involved in crystal packing with the sites revealed by the computational analysis to determine whether predicted druggable sites corresponded to LSD1/CoREST crystal contacts. It was very satisfactory to see (Figure 5) that the regions involved in inter-molecular crystal-packing interactions overlapped closely with both FTMap CSs and SiteMap sites. For instance, the Tower domain had minimal SiteMap and FTMap hot spots. Nevertheless, the crystal-contact inspection showed that the Tower of an LSD1/CoREST molecule interacted through crystal-contacts with a SiteMap-predicted hot spot on the amine oxidase domain (AOD) of a symmetry-related LSD1/CoREST molecule (Panel B in Figure 5). Likewise, the crystal-contact regions between the AOD and Tower/CoREST-SANT2 domain contained SiteMap-predicted hot spots on both partners (Panel C in Figure 5). These results further validated our approach and supported the observation that the identified sites represented promising small-molecule or protein-protein interaction sites.

**Figure 6:**
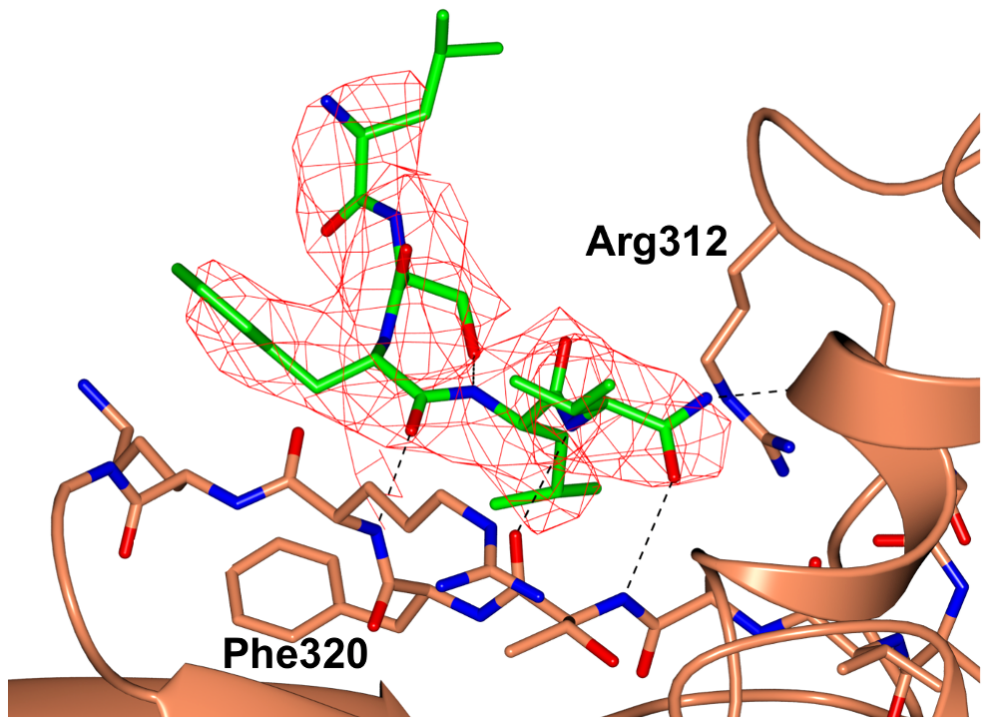
Crystallographic data of Pro-Leu-Ser-Phe-Leu-Val binding to a region on the surface of LSD1/CoREST AOD. The peptide (green) and LSD1 (brown) are highlighted (nitrogen atoms: blue; oxygen atoms: red). The unbiased 2Fo-Fc electron density map (contoured at 1.2 σ level) was calculated prior to inclusion of the peptide in the refinement. Residue Pro1 of the bound peptide was not visible in the electron density and, therefore, was not included in the model. See also Table 1 and Figure 8, region D.

Additional support to the validity of our approach was given by the investigation of the crystal structure of LSD1/CoREST bound to Pro-Leu-Ser-Phe-Leu-Val. This peptide was investigated in the framework of a study aimed at identifying the sequence features that confer specificity to the interaction between the LSD1/CoREST active site and the N-terminal SNAG domain of SNAIL1 and related transcription factors [16], [49]. Interestingly, the crystallographic analysis revealed that this peptide binds not only to the catalytic site but also in a distinct shallow cleft in the AOD domain (Figure 6). The electron density was poorly defined for Pro1, but showed well-defined conformations for all other ligand residues bound to this newly discovered site. In particular, the peptide adopted an extended conformation that enabled its backbone to establish H-bond interactions with an adjacent β-strand (residues 317–323). Furthermore, Phe4 and Val5 were both engaged in van der Waals contacts with nearby residues (Ala318, Thr319, Phe320, Leu329, and Val747). It remains to be seen whether this region actually represents a potential site for interactions between LSD1 and other proteins; this will be the subject of future studies.

**Figure 7:**
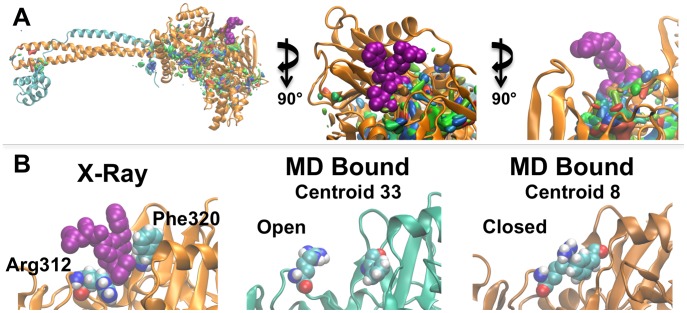
MD reduced ensemble shows SiteMap sites predicted small peptide binding to LSD1 AOD. **Panel A**: SiteMap surfaces from H3-bound MD centroids are mapped onto the structure of LSD1/CoREST bound to Pro-Leu-Ser-Phe-Leu-Val peptide (purple spheres: PDB code 3ZMV). DSV *Visualize* was used to map the SiteMap sites from MD bound centroids onto 3ZMV with SiteMap surfaces colored according to centroid rank. The close-up views show SiteMap sites overlapping with the bound peptide. **Panel B**: Residues Arg312 and Phe320 are key for Pro-Leu-Ser-Phe-Leu-Val peptide binding and SiteMap predictions. Left: The X-ray conformation of Arg312 and Phe320 (Figure 6); SiteMap failed to predict a hot spot in this region after removal of the peptide coordinates. Middle: Instead, Arg312 and Phe320 adopted conformations that opened the Pro-Leu-Ser-Phe-Leu-Val binding pocket leading to SiteMap predictions of a clear hot spot. Right: MD snapshots in which residues Arg312 and Phe320 adopted conformations that closed binding site and prevented the prediction of SiteMap hot spots in the Pro-Leu-Ser-Phe-Leu-Val binding site.

In the context of this work, it was most significant that the peptide-binding site was correctly identified by our computational analysis and showed that including LSD1/CoREST dynamics was crucial. In more detail, neither FTMap nor SiteMap identified this region as a potential hotspot when the crystallographic coordinates were used. However, when the calculations were performed using the LSD1/CoREST configurational ensemble generated from MD snapshots the binding site was correctly located by FTMap on one centroid and by SiteMap on 71% of the centroids (Figure 7A). Examination of the correlation between SiteMap hot spot prediction with specific protein conformational changes highlighted the importance of Arg312 and Phe320 (Figures 6 and 7). During the MD simulations, these residues sampled conformations that enabled SiteMap to identify the region as potential binding site (Figure 7B, second column). Interestingly, Arg312 and Phe320 also sampled configurations that closed the binding pocket and led to negative SiteMap predictions (Figure 7B, third column). These results underscored the importance of including ensembles of LSD1/CoREST structures for exploring the presence of new binding regions even if peptide binding does not cause *per se* any conformational change as gathered by the comparison of the bound and unbound crystal structures. Our findings were in line with a recent study by Johnson and Karanicolas indicating that druggable protein interaction sites are more predisposed to surface pocket formation compared with the rest of the protein surface [54]. On the other hand, it remains to be validated whether all new binding regions identified are favorable binding sites for small drug-like molecules; as suggested by Eyrisch and Helms transient pocket formation on protein surfaces may not be relevant in the context of protein-protein interactions [55]. Ongoing computational and experimental studies are being performed to target the newly predicted regions to discover new molecular probes.

## Discussion

**Figure 8:**
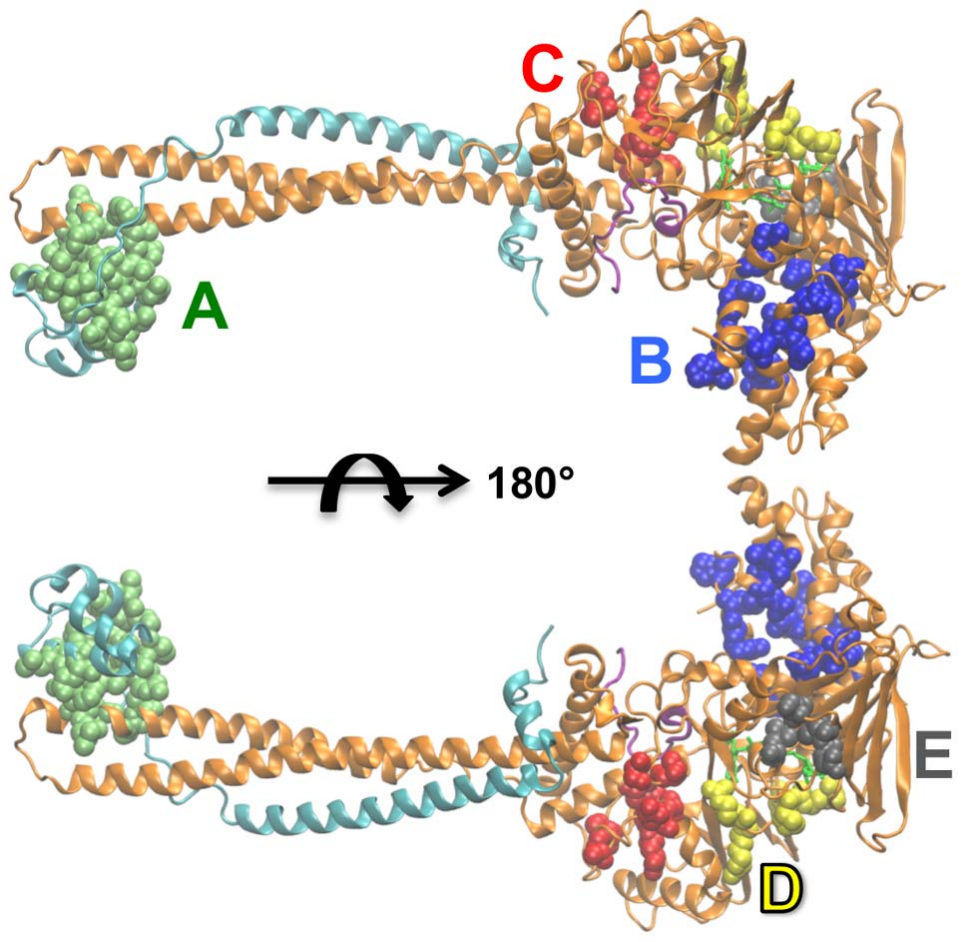
Potential small-molecule and peptide LSD1/CoREST druggable sites. The five most favorable druggable regions thus far unexplored are highlighted using colored spheres and named based on their location: SANT2/Tower interface (green, A), AOD/Tower interface (red, B), SWIRM/AOD interface (blue, C), Peptide binding region (yellow, D), and small AOD pocket (grey, E). LSD1 (orange), CoREST (cyan), and the H3-histone N-terminal tail (purple) are shown as cartoons. Table 1 summarizes the residues in each identified druggable region. See also Figure S2 for a description of the *Select-residues* criterion used for analysis.

An ensemble approach was designed to explore the druggability of dynamic protein receptors and applied to the LSD1/CoREST epigenetic target. Overall, five well-distinct, new binding regions were revealed and display hot spot properties comparable to the well-known H3-histone site (Figure 8). The regions at the SANT2/Tower interface (region A) and at the SWIRM/AOD interface (region B) overlap with the most prominent hinge points revealed by molecular dynamics simulations [17], [18]. We suggest that they could be of primary relevance for LSD1/CoREST chromatin binding. A third interface region overlapping with a dynamic hinge point was discovered at the AOD/Tower interface (region C). These first three regions are optimal targets for the discovery of molecular probes that might block LSD1/CoREST dynamics and prevent chromatin and/or protein association. Supporting experimental evidence of these computationally predicted properties can be obtained by examination of the LSD1/CoREST crystal contacts (Figure 5). A fourth region encompassing the back of the AOD domain was also predicted to have strong propensity for molecular binding (region D). The computational prediction of this region was validated by X-ray crystallography experiments that used small peptides designed to investigate protein-protein interactions on the LSD1/CoREST surface. The co-crystallized Pro-Leu-Ser-Phe-Leu-Val peptide in a novel, blindly predicted binding site on LSD1/CoREST shows the strength of the approach presented. In addition, the observation that this true prediction would be prevented when using only the X-ray structures available (including the structure bound to the same peptide) underscores the relevance of including protein dynamics in the prediction of protein interactions. A fifth region was highlighted corresponding to a small pocket on the AOD domain (region E). On the basis of our molecular dynamics simulations we propose that this predominantly hydrophobic pocket could be relevant as an allosteric site to hamper substrate binding. This study sets the basis for future virtual screening campaigns targeting the five novel regions reported and for the design of LSD1/CoREST mutants to probe LSD1/CoREST binding with chromatin and various protein partners. We developed and presented the Druggable Site Visualizer (DSV) that allows treatment of data of large-size protein configurational ensembles; it is freely distributed to the public, and readily transferable to other protein targets of pharmacological interest.

**Table 1:** LSD1/CoREST residues within each predicted druggable region.

Region	Color in Figure 8	LSD1 Residues	CoREST Residues
A, SANT2/Tower	Green	Phe478, Leu479, Lys481, His484, Arg485	Glu387, Leu389, Leu390, Ala391, Gln393, Gln403, Ala404, Ser406, Asp407, Val408, Ile409, Gly410, Asn411, Lys412, Ser413, Val414, Val415, Gln416
B, SWIRM/AOD	Blue	Ser181, Arg182, His185, Ser190, Gln191, Glu192, Ala193, Gln219, Asp223, Gly260, Ile262, Asn263, Ile266, Met342, Val345, Gly818, Arg820, Glu821, Arg824	
C, AOD/Tower	Red	Ala534, Thr542, Ser545, Val681, Ser683, Glu690, Phe692, Ala708, Glu710	
D, Pro-Leu-Ser-Phe-Leu-Val Binding Site	Yellow	Arg312, Thr319, Arg750, Arg752, Asp754	
E, Small AOD Pocket	Grey	Leu630, Gln638, Phe639, Leu643	

## Supporting Information

Figure S1 **Comparison between including and excluding the H3-histone N-terminal tail during FTMap calculations.** FTMap consensus sites (CSs) from LSD1/CoREST X-ray structure (PDB code 2V1D) with H3-histone N-terminal tail excluded (red: 11 CSs) and FTMap CSs with the H3-histone N-terminal tail included (blue: 16 CSs). The presence of H3-histone N-terminal tail results in FTMap CSs finding diverse regions of the receptor (**A**). FTMap predicts the FAD binding pocket as a favorable binding region (**B**).
(TIFF)

Figure S2 **Druggable Site Visualizer (DSV)** ***Select-residues*** **function with various cutoff distances to FTMap consensus sites (CSs) and SiteMap sites.** The *Select-residues* function of DSV identifies and displays all receptor residues within a specified distance of FTMap CSs and SiteMap sites. The displayed residues largely depend on the distance cutoff. For the case of LSD1/CoREST H3-bound MD centroids a 1-Å cutoff selects zero residues (not shown) but 2-Å, 3-Å, and 4-Å cutoffs select increasingly more residues. The results reported in this paper were based on a 3-Å cutoff.
(TIFF)
