# Supplementary figures and images for: Expanding the Druggable Space of the LSD1/CoREST Epigenetic Target: New Potential Binding Regions for Drug-Like Molecules, Peptides, Protein Partners, and Chromatin
Source: PLoS Comput Biol. 2013 Jul 18;9(7):e1003158. doi: 10.1371/journal.pcbi.1003158 (PMC3715402; doi:10.1371/journal.pcbi.1003158)

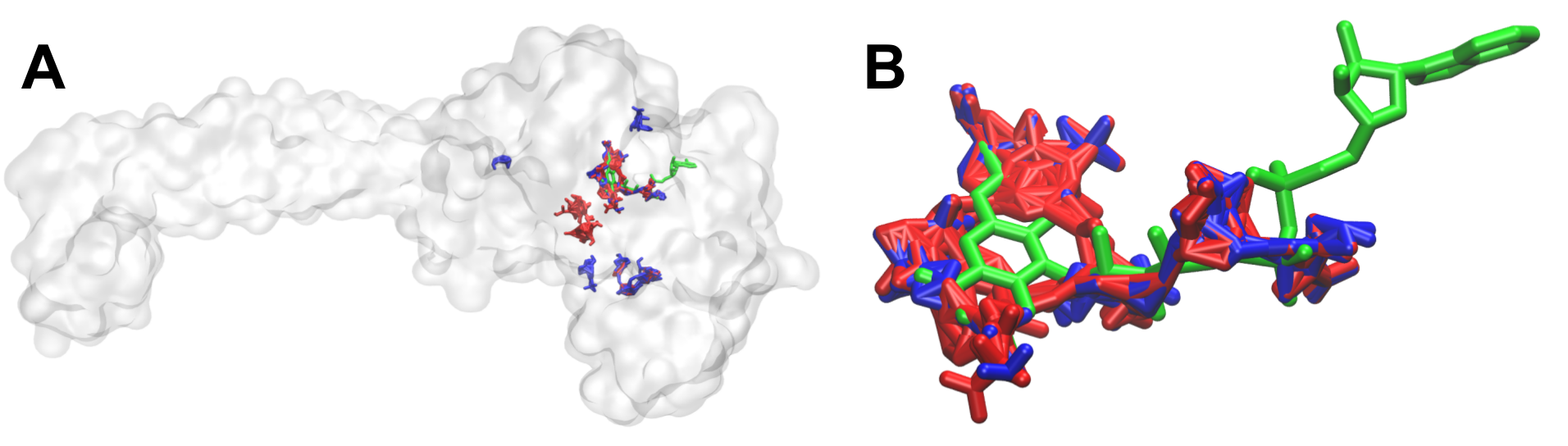

Supplement: Figure S1 — Comparison between including and excluding the H3-histone N-terminal tail during FTMap calculations. FTMap consensus sites (CSs) from LSD1/CoREST X-ray structure (PDB code 2V1D) with H3-histone N-terminal tail excluded (red: 11 CSs) and FTMap CSs with the H3-histone N-terminal tail included (blue: 16 CSs). The presence of H3-histone N-terminal tail results in FTMap CSs finding diverse regions of the receptor (A). FTMap predicts the FAD binding pocket as a favorable binding region (B). (TIFF) [file pcbi.1003158.s001.tiff]

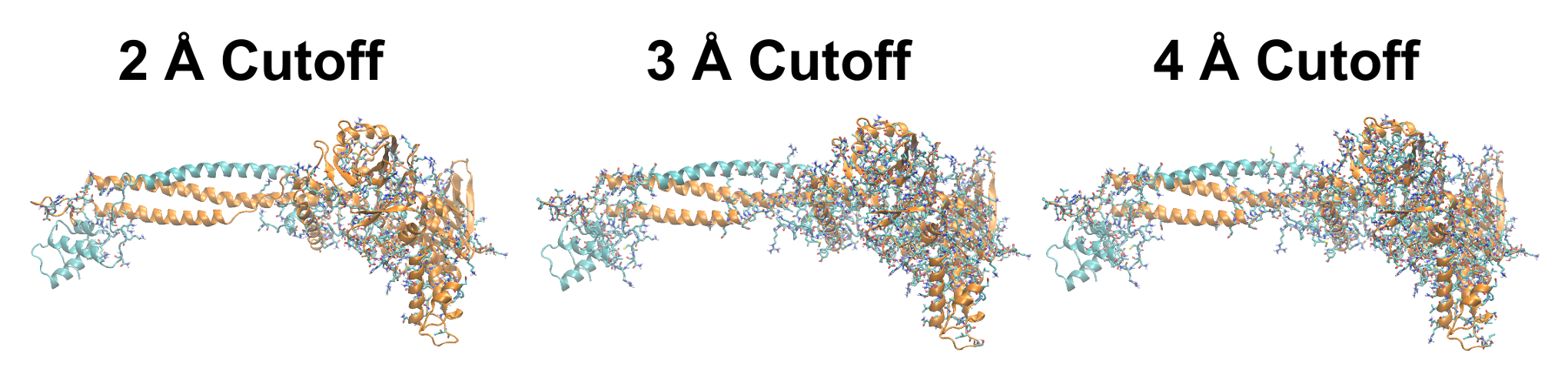

Supplement: Figure S2 — Druggable Site Visualizer (DSV) Select-residues function with various cutoff distances to FTMap consensus sites (CSs) and SiteMap sites. The Select-residues function of DSV identifies and displays all receptor residues within a specified distance of FTMap CSs and SiteMap sites. The displayed residues largely depend on the distance cutoff. For the case of LSD1/CoREST H3-bound MD centroids a 1-Å cutoff selects zero residues (not shown) but 2-Å, 3-Å, and 4-Å cutoffs select increasingly more residues. The results reported in this paper were based on a 3-Å cutoff. (TIFF) [file pcbi.1003158.s002.tiff]
